# Marine Sensors: Recent Advances and Challenges

**DOI:** 10.3390/s23042203

**Published:** 2023-02-15

**Authors:** Luís Gonçalves, Marcos Silva Martins, Rui A. Lima, Graça Minas

**Affiliations:** 1 Center for MicroElectromechanical Systems (CMEMS-UMinho), Campus de Azurém, University of Minho, 4800-058 Guimarães, Portugal; 2 LABBELS—Associate Laboratory, 4800-058 Guimarães, Portugal; 3 INESC TEC, 4200-465 Porto, Portugal; 4 CEFT, Faculdade de Engenharia da Universidade do Porto (FEUP), Rua Roberto Frias, 4200-465 Porto, Portugal; 5 MEtRICs, Mechanical Engineering Department, Campus de Azurém, University of Minho, 4800-058 Guimarães, Portugal

The ocean has a huge impact on our way of life; therefore, there is a need to monitor and protect its biodiversity. Additionally, the ocean industrial potential for health, minerals, and oil has promoted the need for constant and real-time monitoring. Due to all of these concerns, it is important to develop efficient and smart marine sensors to improve our knowledge of the sea environment and to support marine sustainable development.

This Special Issue starts with the development of different devices and sensors for underwater applications in the ocean. Faria et al. [1] developed a linear electromagnetic energy harvesting device for underwater applications to be operated with movement frequencies from 0.1 to 0.4 Hz. This demonstrated that this energy is sufficient to restore energy used by the battery or the capacitor and continue supplying energy to the sensors. Dyomin et al. [2] developed an underwater holographic sensor to study marine particles, whereas Martins et al. [3] developed and characterized a polyvinylidene difluoride ultrasound transducer to be used as an emitter in underwater wireless communications. In two other articles related to this topic, Nguyen et al. [4] classified underwater sonar images using convolutional neural networks to detect a submerged human body, whereas Sheng et al. [5] proposed a bioinspired twin-inverted multiscale matched filtering method to detect underwater moving targets.

In addition to those articles, this Special Issue also presents the development of optical sensors for marine environments. Matos et al. [6] developed a cost-effective optical sensor for the continuous in situ monitoring of turbidity and suspended particulate matter concentration, whereas Penso et al. [7] developed and characterized a low-cost and highly sensitive dissolved oxygen optical sensor based on a membrane of PDMS doped with platinum octaethylporphyrin.

Subochev et al. [8] proposed a laser optoacoustic method for the complex characterization of crude oil pollution on the water surface by the thickness of the layer, the speed of sound, the coefficient of optical absorption, and the temperature dependence of the Grüneisen parameter. Li at al. [9] present a paper where they provide a guidance and reference for the in-orbit design of an array orientation for an interferometric microwave radiometer (IMR). Another interesting study was performed by Wang et al. [10], where they proposed a microfluidic system that comprised microalgae cell separation, treatment, and viability characterization. The proposed microfluidic separation system is based on the principle of deterministic lateral displacement (DLD), which can separate various microalgae species rapidly by their different sizes.

Zhang et al. [11] developed an adaptive waveform design for cognitive radar for extended target detection under compound-Gaussian (CG), whereas Höschle et al. [12] showed the potential in which very high-resolution satellite imagery might be used to address urgent questions in whale conservation.

The final two papers are studies that can be applied in different kinds of lakes. Chen et al. [13] evaluated the feasibility of the vicarious calibration method at the Lake Qinghai RCS, whereas Aslamov et al. [14] proposed an autonomous monitoring system for the continuous in situ measuring of vertical temperature distribution in the near-ice air, ice strata, and under-ice water layer for several months with simultaneous records of solar radiation incoming at the lake surface and passing through the snow and ice covers, as well as snow and ice thicknesses.

As Guest Editors, we hope that this book can provide an opportunity for the engineering and biological marine community to acquire knowledge and information on the latest advances and challenges in marine sensors and their applications to monitor sea and lake environment.

On a final remark, we would like to thank all the authors for contributing their original manuscripts to this Special Issue, as well as the reviewers for spending their precious time in the review process, a task essential to improve the quality of the submitted papers.
